# Fente palatine négligée traitée au Centre hospitalier universitaire pédiatrique de Bangui (République centrafricaine)

**DOI:** 10.48327/mtsi.v3i2.2023.377

**Published:** 2023-05-30

**Authors:** Daniel Sylvère OUAÏMON, Valère NDOMA NGATCHOUKPO, Jacob Israël Junior SOPIO, Arnaud Rodrigue BOROHOUL

**Affiliations:** 1 Centre hospitalier universitaire pédiatrique de Bangui (CHUPB), République centrafricaine; 2 Faculté des sciences de la santé de l'université de Bangui, République centrafricaine

**Keywords:** Fente palatine, Adolescente, Dépistage, Prise en charge, République centrafricaine, Afrique subsaharienne, Cleft palate, Adolescent, Screening, Management, Central African Republic, Sub-Saharan Africa

## Abstract

La fente palatine est l'absence de substance de la voûte buccale aboutissant à une communication entre le nez et la bouche. Il s'agit d'une malformation congénitale fréquente dont les facteurs étiologiques incriminés sont exogènes et génétiques. Le diagnostic est clinique et la recherche des étiologies repose généralement sur des analyses de sang. La prise en charge de la fente palatine s'organise dorénavant dans un cadre multidisciplinaire où la coordination de chaque spécialité permettra d'harmoniser les différents traitements. L'attitude actuelle repose sur le principe d'une prise en charge précoce permettant de restaurer le plus rapidement possible la fonctionnalité des sangles musculaires vélopalatines réduisant les risques de séquelles phonatoires et auditives. Concernant les fentes palatines, les données sont parcellaires et quasi inexistantes en République centrafricaine, d'où l'intérêt de mettre à la lumière du jour ce cas clinique présent chez une adolescente de 13 ans découverte au décours d'une campagne de dépistage et traitement des fentes labiopalatines au Centre hospitalier universitaire pédiatrique de Bangui.

## Abstract (en)

Cleft palate is the absence of the vault of the mouth resulting in communication between the nose and the mouth. The current frequency of cleft is 0.3% among black people. The incriminated etiological factors of this congenital malformation are exogenous and genetic. The diagnostic is clinical and above all marked by the consequences linked to the cleft, which are multiple morphological problems entailing difficulties from the first days of life (sackling impossible in the event of total cleft lip and palate), functional disorders linked to cleft velopalatine by bucco-nasal reflux, major phonation disorder, dysfunction of the Eustachian tubes causing hearing and otorhinolaryngology (ORL) disorders, breathing disorders. Thanks to the progress of ultrasound, the diagnosis can sometimes be made during pregnancy. Obstetric ultrasound can diagnose cleft lip and palate from the 16th week of amenorrhea. The Veau-Wardill classification based on embryology makes it possible to understand the different types.

The care is now organized in a multidisciplinary frameware where the coordination of each specialty will make it possible to harmonize the different treatments. The current attitude is based on the principe of early management to restore the functionality of the velopalatine muscle straps as quickly as possible, reducing the risk of phonatory and auditory sequelae.

Concerning cleft palates, the data are fragmented and almost non-existent in the Central African Republic, hence the interest in publishing this clinical case highlighted by chance. We report a clinical case of neglected cleft palate in a 13-year-old girl discovered during a cleft lip and palate screening campaign at the Pediatric University Hospital of Bangui.

## Introduction

Les fentes labiovélopalatines (FLVP) représentent une malformation congénitale fréquente diversement perçue selon les ethnies. Les difficultés de la chirurgie résident autant dans ses indications que dans la réalisation du geste opératoire lui-même. Elles sont d'autant plus importantes qu'il convient de décider d'une attitude thérapeutique souvent précoce mais dont les résultats ne seront évalués que de nombreuses années plus tard [1, 2]. Selon la forme anatomoclinique – labiale, vélaire, vélopalatine ou bien labio-alvéolo-vélo-palatine –, les conséquences fonctionnelles et esthétiques varient, aggravant parfois lourdement le pronostic social de l'enfant. À ces contraintes thérapeutiques spécifiques s'ajoute celle de la prise en charge d’éventuelles pathologies associées nécessitant des compétences souvent variées et multidisciplinaires [4, 5, 6, 7].

Nous rapportons un cas clinique de fente palatine négligée prise en charge par une équipe multidisciplinaire au sein du CHUPB.

## Observation

Il s'agissait d'une adolescente de 13 ans, pesant 48 kg, demeurant à Bangui avec un faible niveau socio-économique qui a consulté dans le cadre de la campagne des fentes labiopalatines en juillet 2021. Les plaintes étaient un trouble de phonation faisant suite à une fente palatine survenue depuis la naissance à l'issue d'une grossesse mal suivie avec deux consultations prénatales. À la 8^e^ semaine de grossesse, la maman avait présenté des roséoles syphilitiques marquées par une éruption généralisée de petites papules sur le tronc et les membres supérieurs, et des adénopathies dans les aires ganglionnaires inguinales. Après examen, elle était classée à la 2^e^ phase ou syphilis secondaire. Une sérologie syphilitique positive chez la mère, à savoir TPHA+ *(Treponema pallidum* Hae-magglutination Assay) 13255, VDRL+ (Venereal Disease Research Laboratory) 1528 avec IgM positif, toxoplasmose et rubéole négatives. L'accouchement était à 37 semaines d'aménorrhée, par voie basse.

On notait sur le nouveau-né des ulcérations cutanées, des adénopathies inguinales, des fissures péribuccales. Placenta et cordon ombilical étaient normaux, l'analyse du sang et du prélèvement cutané chez le nouveau-né a retrouvé le test IgM positif ainsi que le test TPHA positif. La mère avait bénéficié d'un traitement à base de doxycycline 100 mg 1 cp matin et soir pendant 14 jours, et le nouveau-né par pénicilline G 50 000 unités/kg IV toutes les 12 heures pendant 10 jours. Un contrôle de la charge VDRL et TPHA réalisé chez la mère à 3, 6 et 12 mois était satisfaisant.

**Figure 1:**
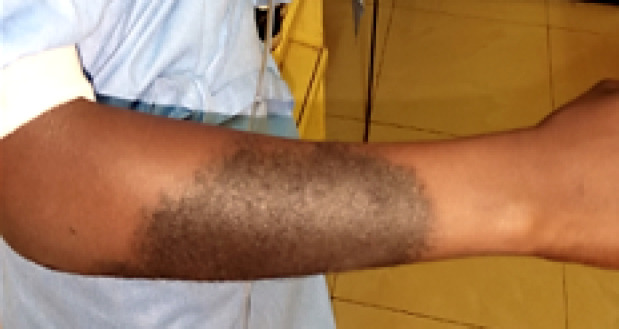
Lésion cutanée congénitale (crédit photo: Daniel Sylvère Ouaïmon, CHUPB) Congenital skin injury (photo credit: Daniel Sylvère Ouaïmon, CHUPB)

**Figure 2:**
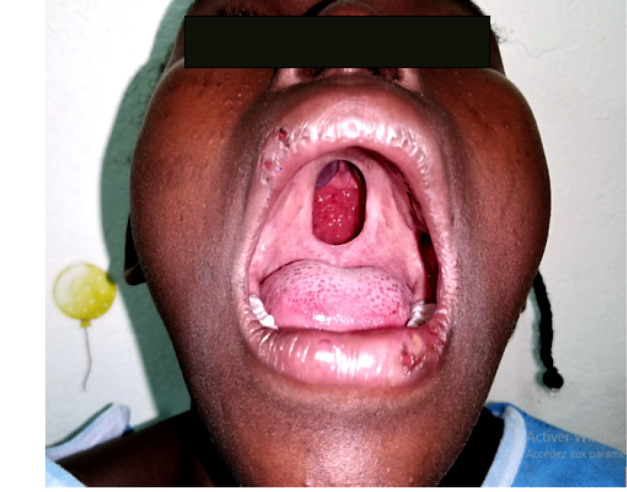
Fente palatine isolée (crédit photo: Daniel Sylvère Ouaïmon, CHUPB) Isolated cleft palate (photo credit: Daniel Sylvère Ouaïmon, CHUPB)

**Figure 3:**
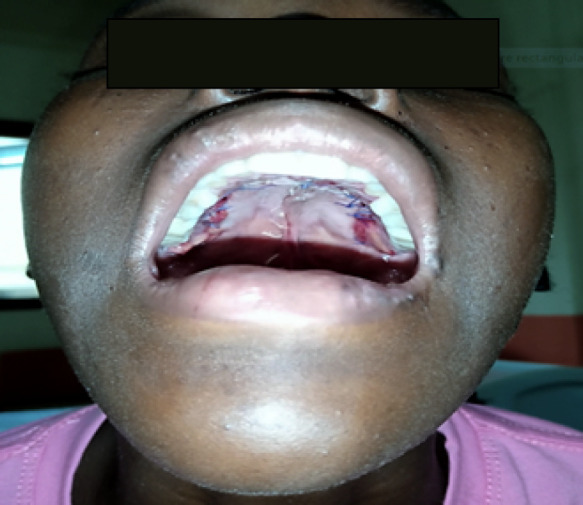
Fente palatine opérée (crédit photo: Daniel Sylvère Ouaïmon, CHUPB) Cleft palate surgery (photo credit: Daniel Sylvère Ouaïmon, CHUPB)

**Figure 4:**
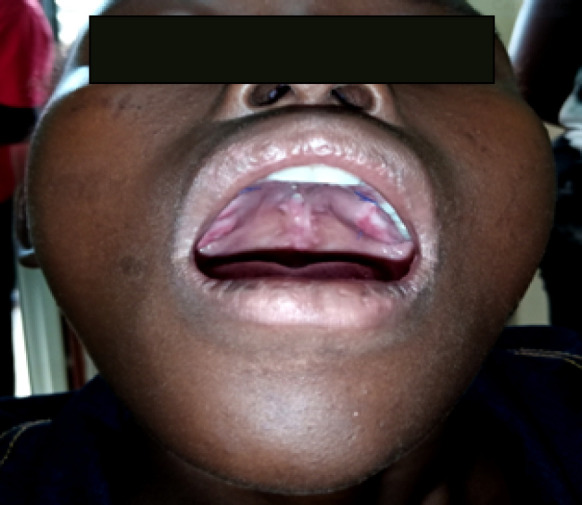
Plaie après 4 mois de cicatrisation dirigée (cicatrice rétractile) Wound after 4 month of directed healing (retractile scar)

À ce jour, l'examen clinique chez notre patiente a mis en évidence un bon état général, une pression artérielle à 120/70 mm Hg, une fréquence respiratoire à 18 cycles/mn, une température de 37, 7 °C, un poids de 38 kg. L'examen physique a retrouvé une plaque veloutée noircie au niveau de l'avant-bras droit dont le plus grand diamètre faisait 17 cm, aux limites bien définies, associée à des poils centraux et périphériques (Fig. 1). Les aires ganglionnaires étaient libres. L'inspection de la cavité buccale à l'aide d'un abaisse-langue permettait de voir une fente palatine isolée, associée à une fistule congénitale postérieure du palais, et un trouble de la phonation (Fig. 2). L'examen des autres appareils était sans particularités, pas d'autres malformations objectivées. Le bilan infectieux réalisé sur elle retrouvait une sérologie TPHA+, VDRL- et la sérologie VIH après consentement était négative. L’équipe multidisciplinaire a décidé de l'opération par une staphylorraphie selon la technique de Veau-Wardill avec lambeau graisseux, qui consistait à créer des lambeaux muco-périostés palatins de part et d'autre de la fente, avec tracé de refend antérieur en V. La suture par translation interne de chaque hémi-voile a été réalisée avec du fil résorbable 5/0. Le tampon monté a été retiré à la fin de l'intervention et les suites opératoires ont été simples, marquées par une antibiothérapie et un anti-inflammatoire, la reprise de l'alimentation 24 h après (Fig. 3). Le recul après 180 jours montrait une bonne cicatrisation sans fistule et une amélioration nette de la phonation (Fig. 4).

## Discussion

Notre patiente était en classe de 6^e^ au lycée et éprouvait beaucoup de difficulté à s'exprimer car elle avait un trouble de phonation. Selon son entourage, elle était timide et n'adressait pas la parole aux autres à cause de son handicap. Devant la réaction négative de ses camarades, elle s'est isolée et fréquentait peu les gens. Son état pourrait être expliqué par la présence de cette fente palatine congénitale avec fistule sans handicap intellectuel. Après la chirurgie, elle a présenté une phonation acceptable et a retrouvé ses camarades. Les fentes labiopalatines sont les malformations congénitales les plus fréquentes au niveau du visage et de la bouche. Elles sont dues à un défaut de soudure des bourgeons embryonnaires au cours des premières semaines de l'embryogenèse [9]. Une prédisposition héréditaire peut être mise en cause mais aussi certaines influences environnementales néfastes, ou la prise d'un agent supposé tératogène comme les antiépileptiques ou une intoxication éthylique chronique [8, 9]. Notre patiente a un niveau de vie socio-économique bas, donnant à penser que son environnement a affecté son état. Le diagnostic des FLVP est clinique et surtout marqué par les conséquences liées à la fente [10]. Grâce au progrès de l’échographie, le diagnostic peut être posé pendant la grossesse à partir de la 16^e^ semaine pour la fente palatine [11].

Dans notre cas, une syphilis secondaire avait été diagnostiquée chez la mère à la 8^e^ semaine d'aménorrhée, ce qui pourrait être la source des malformations [8, 9, 10, 11]. La syphilis vénérienne (*T. pallidum pallidum*) est répandue dans le monde entier, mais son incidence varie en fonction des emplacements géographiques et des groupes socio-économiques. En Afrique, le taux de positivité des tests sérologiques de dépistage de la syphilis dans les consultations prénatales oscille entre 3,6 et 19% [9]. Il est de 7,6% en Centrafrique ce qui s'explique par des rapports sexuels majoritairement non protégés ainsi que la réhabilitation des différents axes routiers et le flux migratoire. La maladie est transmise à partir du 4^e^ mois de gestation avec risque d'avortement, d'accouchement prématuré et de mort-né. La syphilis endémique évolue elle aussi en différentes phases primaire, secondaire et tardive [9].

La plaque veloutée noircie au niveau de l'avant-bras droit était sans rapport avec la fente palatine et ne peut être attribuée à une syphilis congénitale. Le traitement des fentes palatines est basé sur la reconstruction des trois plans vélaires (nasal, musculaire, mu-queux). L'intervention chirurgicale peut se faire en un ou deux temps. L’âge de l'intervention se situe entre 3 et 6 mois pour les fentes labiales et entre 9 et 12 mois pour les fentes palatines [11, 12, 13]. La staphylorraphie permet de reconstituer le voile du palais long, mobile, avec la possibilité, lors de la contraction, de fermer totalement la communication oro-nasale.

## Conclusion

La chirurgie des FLVP s'inscrit dans le cadre d'une prise en charge globale dont la difficulté est d'obtenir des résultats esthétique et fonctionnel satisfaisants tant immédiatement qu’à distance une fois la croissance du massif terminée.

## Contributions Des Auteurs

Conception et rédaction du manuscrit: Daniel Sylvère OUAÏMON

Discussion et relecture du manuscrit: Valère NDOMA NGATCHOUKPO, Jacob Israël Junior SOPIO, Arnaud Rodrigue BOROHOUL

## Liens D'intérêts

Les auteurs ne déclarent aucun conflit d'intérêts.
